# Plasma-Catalytic Reforming of Naphthalene and Toluene as Biomass Tar over Honeycomb Catalysts in a Gliding Arc Reactor

**DOI:** 10.1021/acssuschemeng.2c02495

**Published:** 2022-06-30

**Authors:** Danhua Mei, Shiyun Liu, Jale Yanik, Gartzen Lopez, Martin Olazar, Zhi Fang, Xin Tu

**Affiliations:** † College of Electrical Engineering and Control Science, Nanjing Tech University, Nanjing 211816, Jiangsu, China; ‡ Department of Electrical Engineering and Electronics, University of Liverpool, Liverpool L69 3GJ, U.K.; § Department of Chemistry, Faculty of Science, Ege University, 35100 Bornova, Izmir, Turkey; ∥ Department of Chemical Engineering, University of the Basque Country UPV/EHU, P.O. Box 644, E48080 Bilbao, Spain; # IKERBASQUE, Basque Foundation for Science, 48013 Bilbao, Spain

**Keywords:** gliding arc, honeycomb catalyst, biomass gasification, tar reforming, plasma catalysis

## Abstract

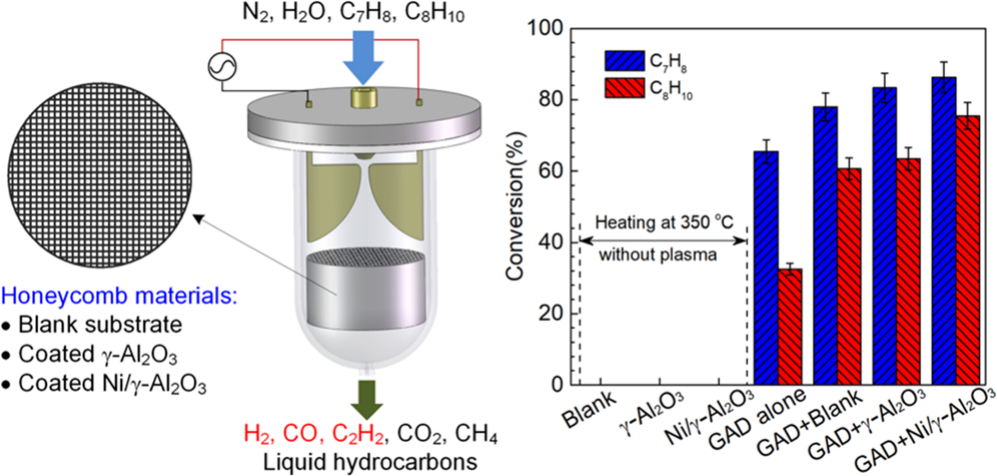

Biomass gasification is a promising and sustainable process to produce renewable and CO_2_-neutral syngas (H_2_ and CO). However, the contamination of syngas with tar is one of the major challenges to limit the deployment of biomass gasification on a commercial scale. Here, we propose a hybrid plasma-catalytic system for steam reforming of tar compounds over honeycomb-based catalysts in a gliding arc discharge (GAD) reactor. The reaction performances were evaluated using the blank substrate and coated catalytic materials (γ-Al_2_O_3_ and Ni/γ-Al_2_O_3_). Compared with the plasma alone process, introducing the honeycomb materials in GAD prolonged the residence time of reactant molecules for collision with plasma reactive species to promote their conversions. The presence of Ni/γ-Al_2_O_3_ gave the best performance with the high conversion of toluene (86.3%) and naphthalene (75.5%) and yield of H_2_ (35.0%) and CO (49.1%), while greatly inhibiting the formation of byproducts. The corresponding highest overall energy efficiency of 50.9 g/kWh was achieved, which was 35.4% higher than that in the plasma alone process. Characterization of the used catalyst and long-term running indicated that the honeycomb material coated with Ni/γ-Al_2_O_3_ had strong carbon resistance and excellent stability. The superior catalytic performance of Ni/γ-Al_2_O_3_ can be mainly ascribed to the large specific surface area and the *in situ* reduction of nickel oxide species in the reaction process, which promoted the interaction between plasma reactive species and catalysts and generated the plasma-catalysis synergy.

## Abstract 2

This work provides a green and sustainable approach for converting biomass gasification tar into valuable chemicals based on plasma chemistry.

## Introduction

The depletion of fossil resources and environmental problems associated with significant greenhouse gas emissions have promoted the development of renewable energy utilization technologies.^1,2^ Biomass is considered a renewable carbon-neutral energy source. Gasification represents an attractive avenue to convert biomass into clean producer gas (a gas mixture of CO, H_2_, CO_2_, and CH_4_). The producer gas is ideally suitable to be utilized in gas turbines and fuel cells to produce heat and electricity or upgraded to synthesize value-added chemical compounds.^3^ However, tar is inevitably formed in gasification, which contains complicated organic compounds, including multiple ring aromatic compounds, and some oxygen-containing hydrocarbons.^4^ The content of tar typically varies between 0.5 and 100 g/m^3^ depending on the type of gasifier.^5^ The presence of tar can cause serious hazards to the end-user devices such as fouling, clogging, and corrosion, lowering the gasification efficiency, as well as increasing the maintenance frequency and the operation cost.^6^ Therefore, effective control and removal of tar is the main challenge to the practical application of producer gas with high efficiency.

Significant efforts have been directed toward tar removal from producer gas using a variety of physical and chemical approaches.^5,7,8^ Mechanical separation mainly removes tar physically using scrubbers, cyclones, and filters. This process is commonly used due to its easy application but will generate secondary pollution and lose the chemical energy contained in tar compounds.^5^ Thermal cracking and catalytic reforming can recycle the energy contained in tar while removing it.^7^ However, transforming tar by thermal cracking normally requires a high reaction temperature of around 1250 °C, which increases the requirement of the reactor and therefore both the capital and operational costs.^8^ Catalytic reforming of tar can achieve promising tar conversions at relatively lower temperatures around 500 °C and high-quality producer gas.^9^ A variety of catalysts have been investigated for catalytic tar reforming, including transition-metal catalysts (Ni, Mn, Fe, and Co), noble-metal catalysts (Pt, Ru, and Rh), and natural catalysts.^10−12^ Among them, Ni-based catalysts have been extensively investigated for tar reforming due to their high reactivity and dehydrogenation capacity.^13^ However, the conventional catalytic reforming process faces major limitations such as rapid catalyst deactivation induced by coke deposition and sintering at high temperatures.^14^

Nonthermal plasmas (NTPs) offer an effective and sustainable alternative approach for converting tars to syngas and other valuable chemicals at lower temperatures.^15,16^ Compared to conventional thermal cracking and catalytic processes, NTP shows unique characteristics of high activity and fast reaction rate, which overcomes the limitation of high reaction temperature and reduces the overall energy cost.^17^ However, the industrial applications of this technology are limited due to low selectivity toward the specific products and the generation of byproducts.^18^ To deal with this issue, the hybrid plasma-catalysis technology has shown great potential as it can combine the advantages of the fast reaction rate of NTPs and the high selectivity of the catalyst with the high activity.^19−22^ The synergistic effect might be generated in the hybrid plasma-enhanced catalytic system, where the catalysts can be activated at low temperatures with high reactivity and strong carbon resistance.^23−26^ Currently, dielectric barrier discharge (DBD) has attracted intense attention in the plasma-catalytic reforming of biomass tars as it has strong flexibility to be combined with catalysts to promote the conversion of tar model compounds and the yield of specific products while suppressing the formation of undesired byproducts.^27−29^ Nevertheless, the energy efficiency of the tar reforming process based on DBD plasma coupled with catalysis is still unsatisfactory due to the limited treatment capability and power levels.

Compared to DBD, gliding arc discharge (GAD) is featured by simple configuration, high processing capacity, and relative higher energy density, which enable it to show more potential for efficient destruction and reforming of tar.^30−34^ Moreover, the enhanced reaction performance could be achieved by introducing suitable catalysts into the GAD reactor, which has been confirmed in CO_2_ conversion and CH_4_ activation using GAD.^35−37^ For the biomass gasification tar, we previously performed the conversion of naphthalene and toluene mixture (model tar compound) in GAD coupled with a Ni-Co/γ-Al_2_O_3_ bimetallic catalyst to obtain the highest total tar conversion (95.1%) and overall energy efficiency (40.3 g/kWh).^38^ Xu and co-workers found that packing a Ni/γ-Al_2_O_3_ catalyst bed 62 mm downstream of an anode in a rotating GAD reactor resulted in a toluene conversion of 91.9%, which was 21% higher than that obtained without using any catalyst.^39^ These previous studies demonstrated the effectiveness and benefits of incorporating catalysts into GAD for biomass gasification tar conversion. However, the catalysts were mainly placed in the GAD reactor in the form of a packed-bed, which would cause high pressure drop and therefore enhance the power for fluid flow,^40^ especially for the conditions of high gas flow rate like that required in GAD. To date, little research has attempted to explore an efficient plasma-catalysis configuration using GAD, which can achieve promising performance with high gas flow rate.

Herein, we performed the plasma-catalytic reforming of biomass gasification tar in GAD coupled with honeycomb catalysts. This kind of catalyst offers unique features of the uniform gas flow distribution, the strong capability of treating gas with large volumes compared to conventional packed-bed catalysts, and the easiness of scaling up for industrial applications.^41^ The effect of different packing materials downstream of electrodes in GAD was evaluated with respect to tar conversions, selectivities and yields of gaseous products as well as energy efficiencies of the hybrid process. Moreover, a plausible reaction mechanism and pathway involved in our systems were discussed based on the results from catalyst characterizations and a comprehensive analysis of liquid and gas products.

## Experimental Section

### Experimental Setup

**Figure 1:**
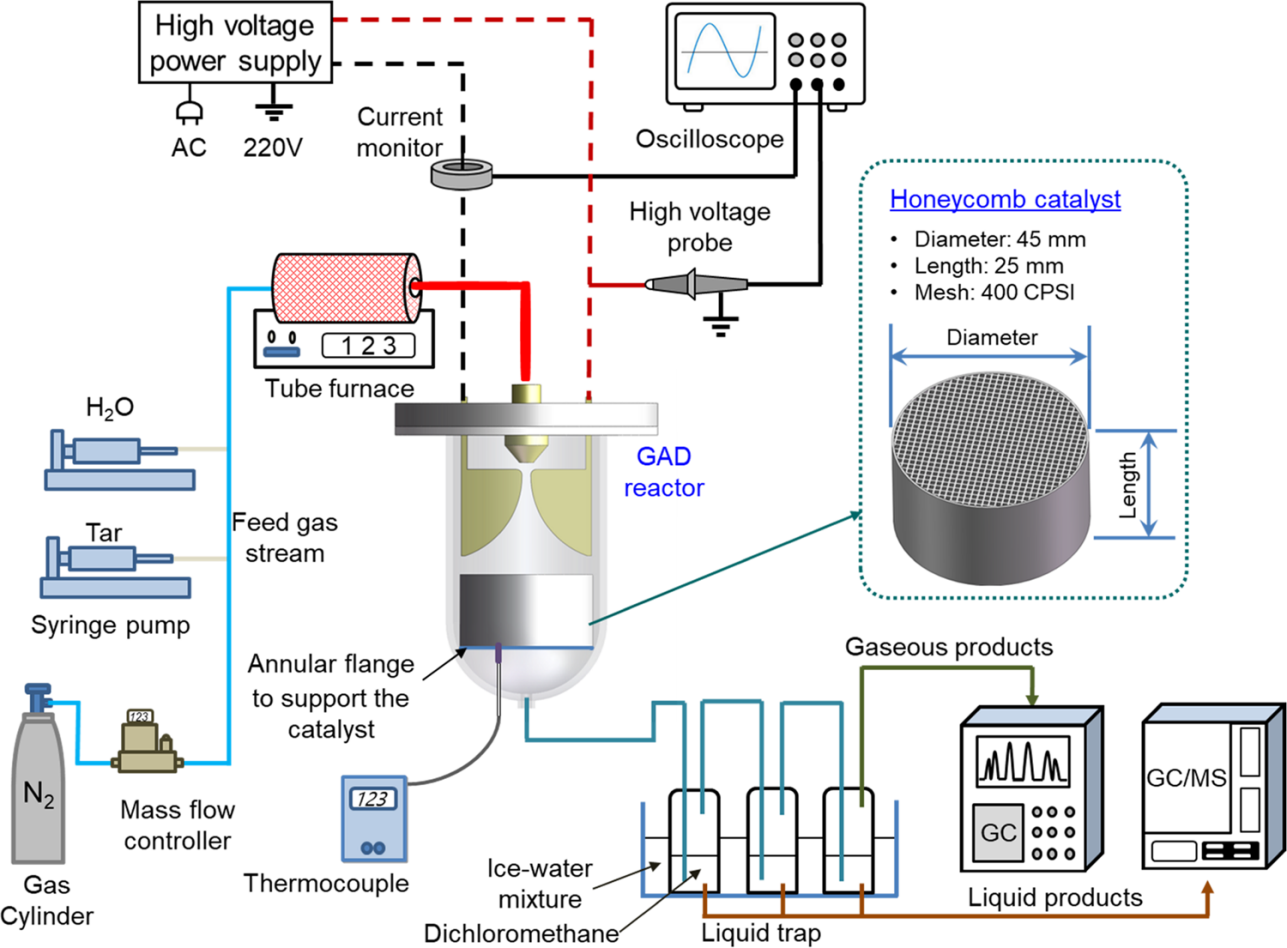
Experimental system for plasma tar reforming.

**Figure 2:**
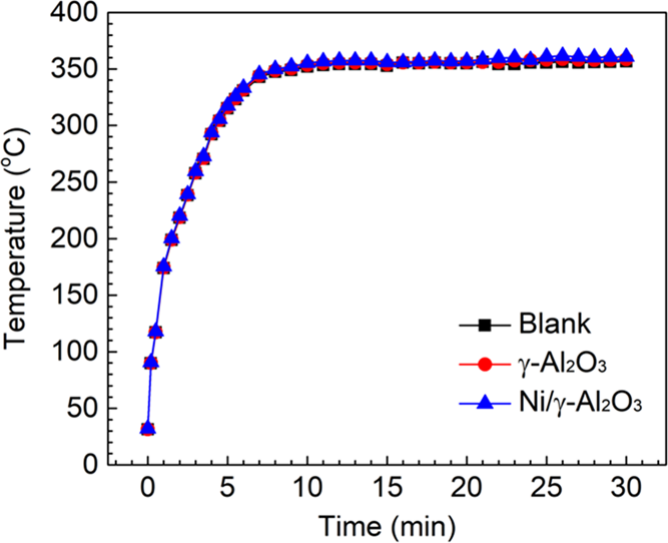
Time evolution of the temperatures in the presence of different honeycomb materials.

The steam reforming of tar was performed in a GAD reactor coupled with honeycomb catalysts (Figure 1). The experimental setup contains a GAD reactor, a carrier gas and reactant supply system, an AC power system, as well as a measurement system for discharge characteristics and reaction performance. The details of the reactor structure and other systems have been presented in our previous studies.^32,38^ A mixture of naphthalene (C_10_H_8_) and toluene (C_7_H_8_) was used as model tar compounds since they represent the typical stable light mono-aromatic and polycyclic aromatic tar compounds from the biomass gasification.^5^ Powders of solid naphthalene were dissolved in toluene to create a mixture of tar compounds. Nitrogen with a high purity of 99.999% was applied as the working gas. Water and the model tar compounds were fed continuously into a gas flow tube using two KDS Legato syringe pumps and evaporated in a tube furnace working at 300 °C. After that, the evaporated mixture was carried to the GAD reactor by the N_2_ flow. The content of naphthalene and toluene in the feed gas was fixed at 1.1 and 15.0 g/Nm^3^, respectively, concerning their amount from the practical biomass gasification process.^30^ The total feed gas flow was kept constant at 3.5 L/min to maintain a stable discharge in the reactor, and the molar ratio of steam-to-carbon was fixed at 1.5. There was no obvious plasma polymerization on the electrodes or reactor walls under these conditions. Similar findings were also reported in our previous works.^30,38^ The plasma reactor was controlled by a 50 Hz neon high-voltage (HV) transformer with an adjustable applied voltage range of 0–10 kV. The discharge power was determined by integrating the applied voltage and arc current, as shown in eq 1. It can be changed by adjusting the applied voltage and was fixed at 56 W for this study.
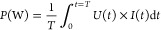
1The packing materials exhibited a honeycomb structure, which was round-shaped with a diameter of 45 mm and a length of 25 mm. The shape of the cell hole in the honeycomb monolith was square with 1 mm sides and cell density was around 400 CPSI (cells per square inch, cell/in^2^). The bare honeycomb substrate was made up of cordierite. γ-Al_2_O_3_ was coated on the substrate and used as the catalyst support. The active metal Ni was then loaded on the catalyst support by the impregnation approach. The bare honeycomb substrate, the catalyst support, and the supported Ni catalyst were all tested in the GAD reactor for biomass gasification tar reforming, and they are denoted as blank, γ-Al_2_O_3_, and Ni/γ-Al_2_O_3_, respectively. The honeycomb materials were placed 2 mm below the electrode end supported by an annular flange during the steam reforming process, as shown in Figure 1. This distance allows the arc to make contact with the catalyst and facilitate the interaction between plasma reactive species and the catalyst, thereby generating the potential plasma-catalysis synergy. The temperatures in the packing materials during the steam reforming process were recorded by a thermocouple. The time evolution of the temperature when using different honeycomb materials is plotted in Figure 2. Clearly, no obvious difference in the temperature was observed in the presence of different honeycomb materials, and they all stabilized at around 350 °C when running the steam reforming reactions for 10 min. We also performed the thermal-catalytic reactions using these three materials at 350 °C to evaluate the plasma-catalysis synergy.

### Catalyst Characterization

The physicochemical properties of the catalysts before and after the reaction were analyzed using the following characterization approaches. The N_2_ adsorption and desorption isotherms of the fresh and used catalysts were analyzed on a Micromeritics ASAP 2020 system. Before the measurement, all prepared samples were vacuum degassed at 150 °C for 5 h to remove the impurities. The pore size and specific surface area of the samples were determined by applying the Brunnauer-Emmett-Teller (BET) method. X-ray diffraction (XRD) patterns were collected using an Empyrean diffractometer with a Mo-Ag radiation source in the range 2θ = 5–80° using a turning speed of 4°/min. The morphologies of materials were examined by scanning electron microscopy (SEM) on JEM-2100F SEM equipment at 15 kV. An energy-dispersive X-ray spectrometer (EDX) was also used for the mapping and analysis of the surface elements. The used catalyst was characterized by thermogravimetric analysis (TGA) in an air flow (20 ml/min) using Netzsch STA-449-F3 TGA equipment. The temperature was increased from 20 to 900 °C at a 10 °C/min heating rate.

### Analytical Methods and the Definition of Parameters

The effluent gases from the reactor were first fed into absorption bottles set inside an ice-water mixture cold trap to collect the condensable products and un-converted reactants. The liquid samples were analyzed using gas chromatography-mass spectrometry (GC/MS, 7820A-5975C, Agilent) equipped with an HP-5 capillary column. The recorded mass spectra were analyzed using the National Institute of Standards and Technology (NIST) library. The gaseous products were sampled using gas bags and analyzed by a Shimadzu 2014 GC equipped with dual detectors.

The conversion (*X*) of model tar compound (C_7_H_8_ and C_10_H_8_) and the yields (*Y*) of main gaseous products including H_2_, CO*_x_*, and C*_x_*H*_y_* were determined using the following equations
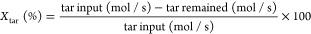
2
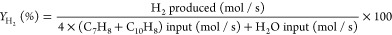
3
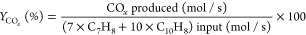
4
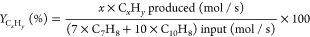
5The selectivities (*S*) of CO*_x_* and C*_x_*H*_y_* were calculated by eqs 6 and 7, respectively.
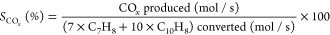
6
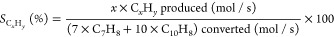
7The energy efficiency (*E*) was defined using eq 8.
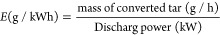
8

## Results and Discussion

### Catalyst Characterization

**Figure 3:**
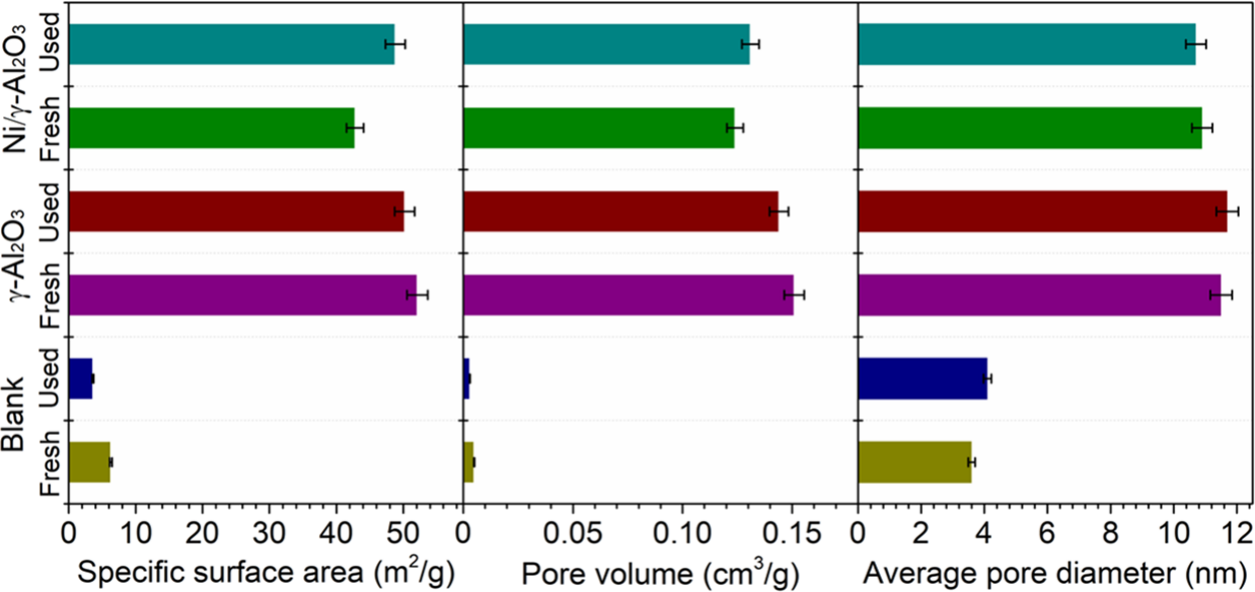
Textural characters of the honeycomb materials.

Figure 3 shows the textural properties of the honeycomb materials before and after the reaction. The blank material exhibited a low specific surface area (*S*_BET_) and small pore size, which can be ascribed to the compact nonporous structure of the bare honeycomb substrate. Coating γ-Al_2_O_3_ on the substrate significantly increased the *S*_BET_ and pore size as γ-Al_2_O_3_ is well known for its high porosity.^42^ After further loading the active metal Ni, the *S*_BET_, average pore diameter, and pore volume slightly dropped, which suggests that the support surface was covered and/or its pores were partially blocked by the active metal.^39^ After the plasma steam reforming reaction, the *S*_BET_ and pore size of the blank material and γ-Al_2_O_3_ were decreased, especially for the blank material, which can be due to the formation of carbon deposition. This was further investigated by TGA analysis. However, the *S*_BET_ and pore size of the used γ-Al_2_O_3_ and Ni catalysts were slightly increased, which suggests that the higher *S*_BET_ for the reaction was obtained by the bombardment of ions and/or electrons produced by the GAD plasma during the steam reforming process.

**Figure 4:**
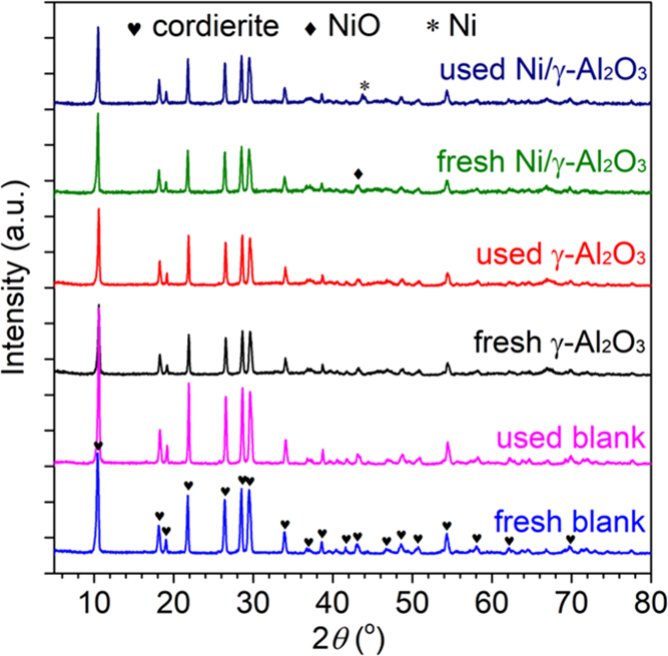
XRD patterns of the honeycomb catalysts.

Figure 4 illustrates the XRD patterns of the honeycomb materials before and after the reaction. Clearly, the diffraction peaks of all of the materials were similar with the major peaks located at 2θ = 10.5, 18.2, 21.7, 26.4, 28.5, 29.5, 33.9, and 54.3°, which corresponded to the typical phase of cordierite.^43^ However, compared with the blank substrate, the intensities of the diffraction peaks of γ-Al_2_O_3_ and Ni/γ-Al_2_O_3_ were obviously reduced, revealing that the crystallinity of the honeycomb materials was decreased and the dispersion was enhanced after loading γ-Al_2_O_3_ and active metal Ni successively.^44^ For the blank substrate, its diffraction peaks presented sharper and stronger intensities after the steam reforming reaction. This finding suggests that the crystallite size was increased during the reaction process, which lowered the specific surface area,^45^ as confirmed by the analysis of their textural properties. A slight increase in the diffraction peak intensities was also observed for γ-Al_2_O_3_. Nevertheless, no discernible difference was detected in the diffraction peaks of Ni/γ-Al_2_O_3_ before and after the reaction, which reveals that this honeycomb material could maintain a relatively stable structure in the reaction process. Moreover, the peaks of NiO and Ni were detected at 43.3 and 44.6° in the diffraction peaks of both fresh and spent Ni/γ-Al_2_O_3_, respectively. This phenomenon reveals that the metal oxide NiO species were reduced to Ni in the reaction process. The collision by the energetic electrons generated in the plasma contributed to the reduction as it could dissociate the Ni–O bond in the metal oxide.^46^ The presence of the metal and metal oxide enhances the surface conductivity in the channels of the honeycomb materials, which is beneficial for the propagation of the plasma along the surface of the channels and provides catalytically active sites for steam reforming of tar.^47^ Moreover, the NiO and Ni diffraction peaks were weak and broad, indicating the high dispersion of the reactive species on the catalyst surface.

**Figure 5:**
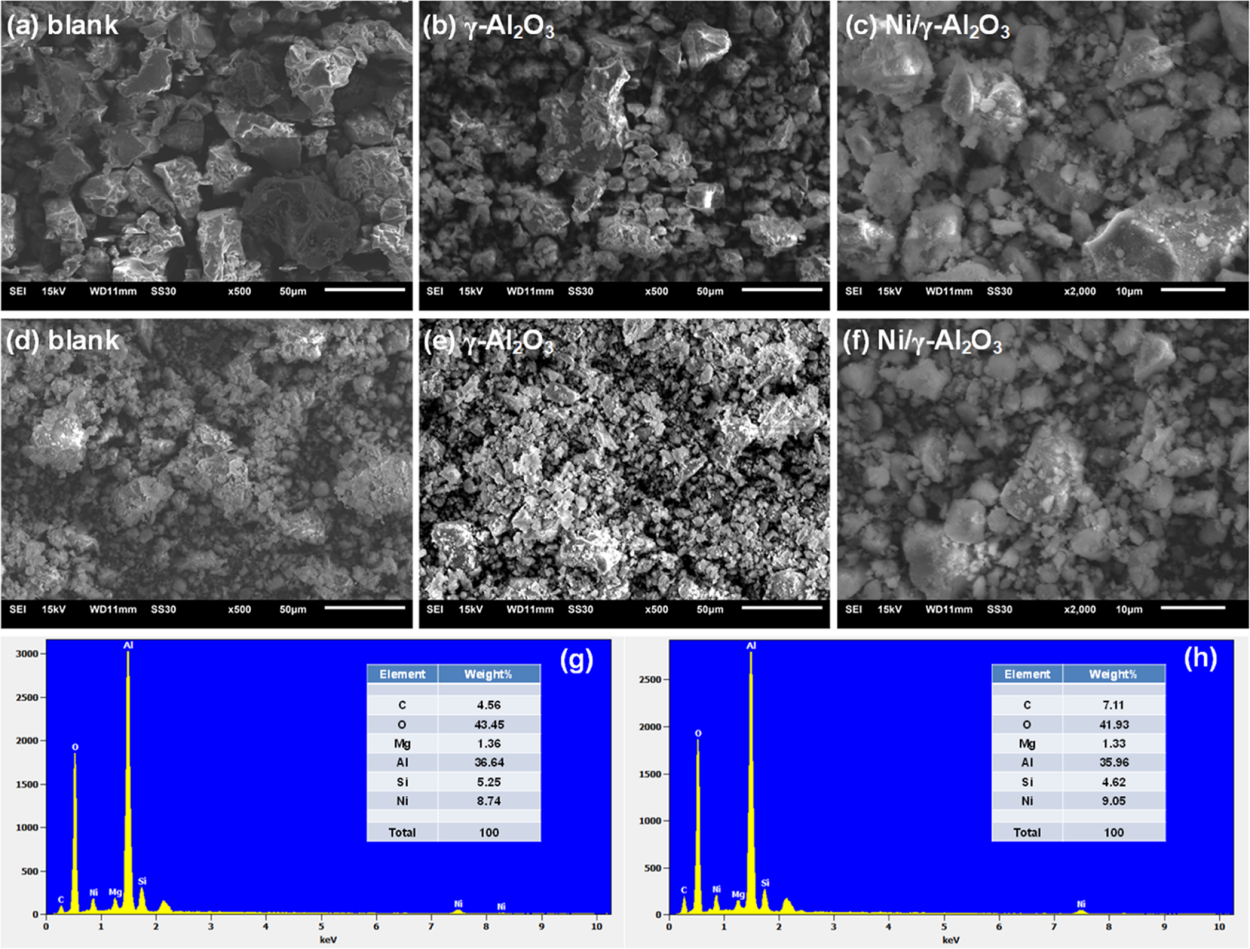
SEM images of blank, γ-Al_2_O_3_ and Ni/γ-Al_2_O_3_ before (a–c) and after (d–f) reaction; EDX graphs of Ni/γ-Al_2_O_3_ before (g) and after (h) reaction.

Figure 5 illustrates the SEM images of all of the fresh and spent honeycomb materials and the EDX graphs of the Ni/γ-Al_2_O_3_ catalyst before and after reaction. Clearly, the surface of the fresh blank substrate was very coarse and contained many cavities (Figure 5a). The crystal grains of γ-Al_2_O_3_ covered the irregular surface of the fresh substrate by coating, which partially filled the cavities and decreased the surface roughness (Figure 5b). The γ-Al_2_O_3_ layer was chemically bonded to the blank substrate and produced a smaller crystallite size, evidenced by the XRD analysis. The relatively uniform metal clusters were attached to the surface of γ-Al_2_O_3_ coated substrate after loading active metal nickel, as shown in the SEM image of Ni/γ-Al_2_O_3_ at higher magnification (Figure 5c). After the plasma reaction, the surface morphologies of the blank material and γ-Al_2_O_3_ were significantly changed due to the production of amorphous and disordered carbon deposition (Figure 5d,e). The deposited carbon might have dissolved into the pores and destroyed these two materials, which decreased their *S*_BET_ and pore volumes. This agrees well with their textural properties in Figure 3. For the Ni/γ-Al_2_O_3_ catalyst, the crystalline structure did not show significant changes and the distribution of active species became more uniform, as shown in Figure 5f. This phenomenon reveals that the GAD plasma contacted with Ni/γ-Al_2_O_3_ promoted the dispersion of Ni species, generating more active sites to interact with tar compounds on the catalyst surface. This positive effect is suggested to come from the bombardment of ions and the attack by the chemically reactive species,^48^ which resulted in the reduction of NiO to Ni as evidenced by the XRD patterns in Figure 4.

The EDX profiles of the Ni/γ-Al_2_O_3_ catalysts are presented in Figure 5g,h. In addition to Ni and Al, the components of cordierite including Mg and Si were detected on the catalyst surface before and after the reaction.^49^ The presence of Mg in the catalyst enhanced the adsorption of steam due to its hydrophilicity, which would lead to a better performance of steam reforming.^50^ After the reaction, the atomic percentage of O was decreased while that of Ni was increased, which also confirmed the reduction of NiO by the plasma active species. The enhanced atomic percentage of C on the spent Ni catalyst suggests the carbon-containing species were deposited on the catalyst surface. This result is in accordance with the TGA analysis.

### Catalytic Performance of the Honeycomb Materials

**Figure 6:**
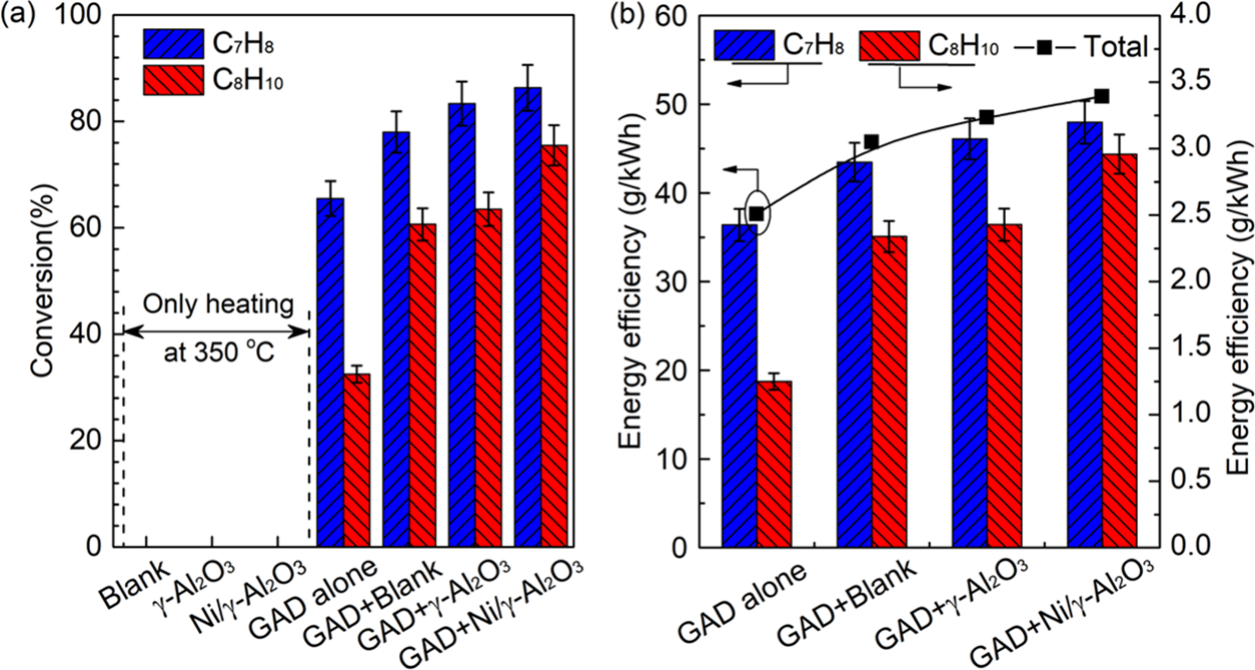
Variations in (a) the tar conversion and (b) the energy efficiency of the plasma reforming under different conditions.

Figure 6 shows the steam reforming performance under different reaction conditions. Clearly, placing the honeycomb materials downstream of the knife-shaped electrode in the reactor substantially increased the reactant conversion and energy efficiency. The maximum conversion of toluene (86.3%) and naphthalene (75.5%) and total energy efficiency (50.9 g/kWh) were achieved using Ni/γ-Al_2_O_3_, which were 31.8, 132.3, and 35.4% greater than those attained during the plasma reaction without a catalyst, respectively. Because naphthalene and toluene have different molecular structures and stability, as well as kinetic reactivity, naphthalene had a lower conversion than toluene under the same operating conditions. This phenomenon was also reported in previous work.^33^ In comparison to toluene, the lower naphthalene content and conversion yielded less converted naphthalene at the same discharge power, thus lowering the energy efficiency for naphthalene conversion. The addition of porous honeycomb materials in the GAD reactor prolonged the residence time of reactants for degradation, which enabled the toluene and naphthalene molecules more susceptible to being attacked by the plasma reactive species and enhanced their conversions. The catalytic performance of the honeycomb materials was basically associated with their *S*_BET_ and pore size.^51^ The material with a higher *S*_BET_ could normally enlarge the contact area for reactant conversion. Loading Ni to the γ-Al_2_O_3_ coated blank substrate slightly reduced the specific surface area but further promoted the energy efficiency and tar conversion, which indicates the core catalytic role of Ni species in the steam reforming reaction. This has been demonstrated in previous studies.^27,39^

The purely thermal-catalytic experiment was performed when the honeycomb materials were heated at 350 °C in the same GAD reactor without discharge to evaluate the function of plasma in the tar reforming reaction (Figure 6a). Clearly, almost no tar compounds were converted in the thermal-catalytic reactions regardless of the honeycomb material type. A comparison of the reaction performance using thermal-catalytic, plasma alone, and plasma-catalytic processes indicates that the performance of the plasma-catalytic system was greater than the sum of that in the thermal-catalytic and plasma alone systems, suggesting the formation of a synergistic effect during the plasma-catalytic process.

**Figure 7:**
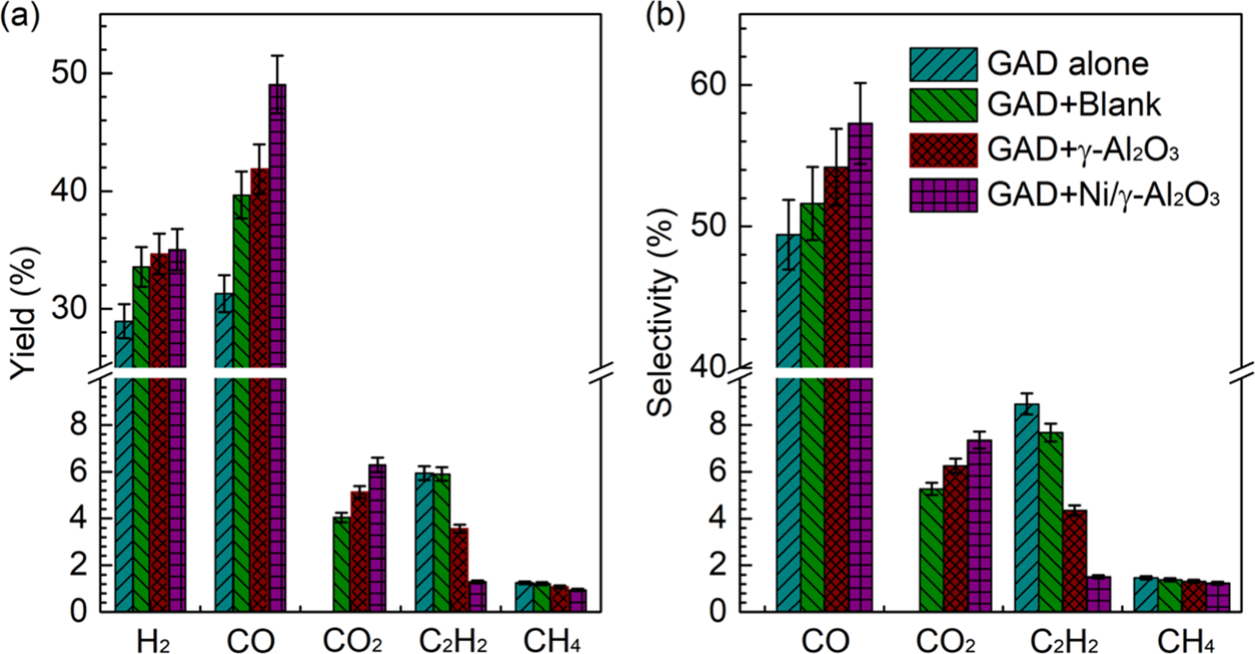
Variations in the (a) yield and (b) selectivity of primary gas products under different conditions.

**Figure 8:**
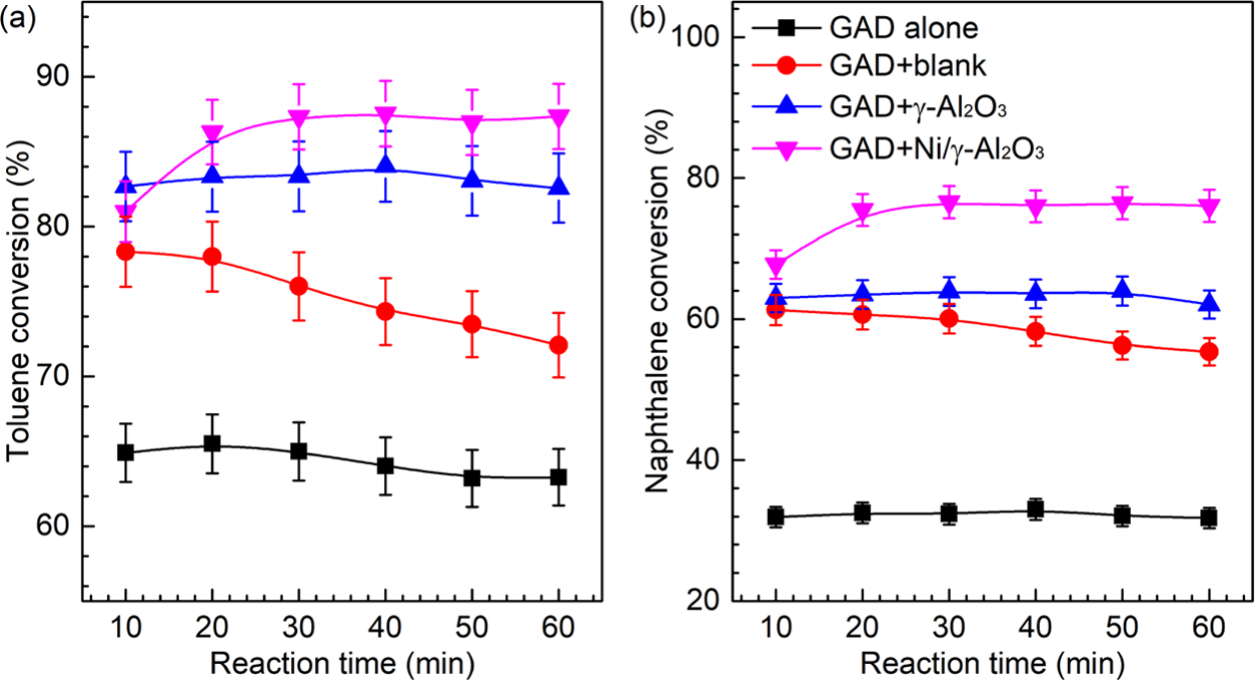
Time variations in the conversions of tar compounds in the different processes.

The yields and selectivities of the gaseous products are displayed in Figure 7. In general, the major gas products consisted of CO, H_2_, CO_2_, C_2_H_2_, and CH_4_ with trace amounts of C_2_H_4_ and C_2_H_6_. Combining the GAD plasma with the honeycomb materials remarkably enhanced the syngas yield, in agreement with the tendency of tar compound conversion. The highest yield of H_2_ (35.0%) and CO (49.1%) was obtained when using Ni/γ-Al_2_O_3_, which was 17.9 and 32.1% greater than that attained using plasma alone, respectively. Integrating Ni/γ-Al_2_O_3_ into the GAD reactor also gave the highest CO selectivity of 57.3%. It is evidenced that CO_2_ was not produced in the plasma alone process, while introducing the honeycomb materials dramatically promoted the formation of CO_2_. The highest yield (6.3%) and selectivity (7.3%) of CO_2_ were obtained when using the supported Ni catalyst. This phenomenon implies that the catalysts under the plasma conditions initiated the water–gas shift reaction (R1) while promoting the steam reforming of tar compounds (R2) due to the accumulation of H_2_O molecules on the honeycomb material surface.^52,53^ This finding was consistent with that reported by Cimerman et al.^54^ They found that the combination of plasma with packing materials (e.g., TiO_2_ and Pt/γ-Al_2_O_3_) for reforming of naphthalene significantly promoted the formation of CO_2_. In addition, the presence of these honeycomb materials inhibited the formation of C_2_H_2_ and CH_4_. The lowest yield and selectivity of these two hydrocarbons were obtained when using the Ni/γ-Al_2_O_3_ catalyst. It has been reported that C_2_H_2_ is prone to be hydrogenated to form C_2_H_4_ and C_2_H_6_ in the presence of metal-supported catalysts under plasma conditions.^55^ This might be the main reason for the decline in the yield and selectivity of C_2_H_2_. CH_4_ is mainly generated from the recombination of H and CH_3_ (R3). In plasma-catalytic reforming process, the CH_4_ decomposition reaction (R4) and CO disproportion reaction (R5) are considered to be the primary pathways for carbon deposition.^18,29^ The use of honeycomb materials increased CO yield and selectivity, indicating that the CO disproportion reaction was less important for carbon deposition in this study. However, the low yield and selectivity of CH_4_ when using the supported Ni catalyst would contribute to the formation of limited carbon on the used catalyst.
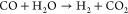
R1
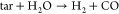
R2
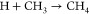
R3
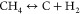
R4
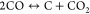
R5Figure 8 displays the time variations in the conversion of naphthalene and toluene under different conditions. The presence of the honeycomb materials exhibited higher reactant conversions compared with the plasma alone process. A significant decline in the reactant conversions with reaction time was observed when using the blank substrate. This might be resulted from the severe carbon deposition due to its lower *S*_BET_. In the reaction using plasma catalysis, the Ni sample was activated with the increasing temperature in the initial 20 min. In addition, the NiO species were reduced to Ni during this stage, as evidenced by the XRD patterns. The reduced metal Ni has been reported to show better activity than its metal oxide NiO in the steam reforming reaction.^46^ These factors contributed to the enhancement in the reactant conversions in the initial stage of the reaction process. In Figure 8, only a slight fluctuation in the reactant conversions was observed when the supported Ni catalyst was fully activated, which implies that the plasma-catalytic process using GAD and the Ni catalyst with honeycomb support showed promising stability.

### Characterization of the Used Honeycomb Materials

**Figure 9:**
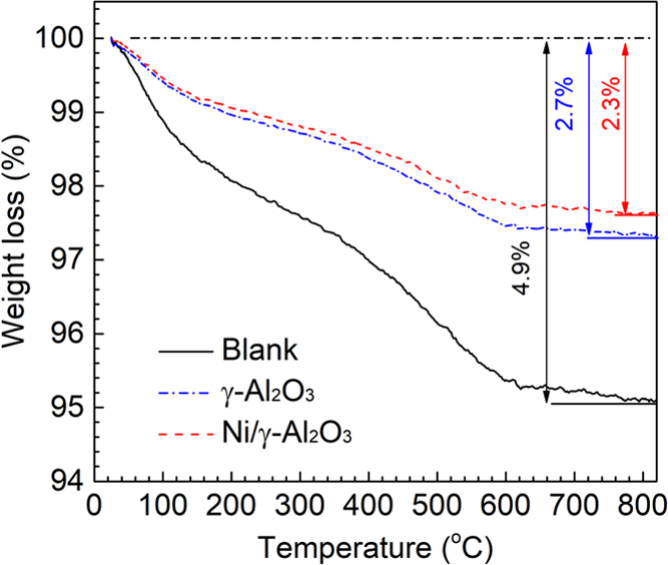
TGA curves of the used honeycomb materials after 60 min plasma reaction.

The used honeycomb materials running the plasma steam reforming process for 60 min were characterized by TGA to estimate the carbon deposition on their surface (Figure 9). The used blank, γ-Al_2_O_3_ and Ni/γ-Al_2_O_3_ exhibited a continuous weight loss over two main steps with a total mass loss of 4.9, 2.7, and 2.3%, respectively. The first weight loss step in the temperature range between 25 and 150 °C represents the evaporation of adsorbed H_2_O. The second weight loss between 150 and 800 °C corresponds to the removal of the deposited carbon. Specifically, the weight loss at ∼200 to 380 °C can be ascribed to the oxidation of amorphous carbon, while that at temperatures higher than 500 °C can be due to the oxidation of graphitic and whisker carbon.^56^ The formation of whisker and graphitic carbon is the main contribution to the catalyst deactivation as they could not be oxidized in the GAD reactor due to the low temperature in the honeycomb materials (around 350 °C). The TGA curve of Ni/γ-Al_2_O_3_ showed the smallest amount of weight loss at 500–800 °C, indicating the outstanding capability to limit the carbon formation on the honeycomb material with the addition of metal elements. This was responsible for its superior performance including tar conversion, yield and selectivity of the primary gaseous products, and reforming efficiency.

### Liquid Byproducts and Mechanisms Analysis

**Figure 10:**
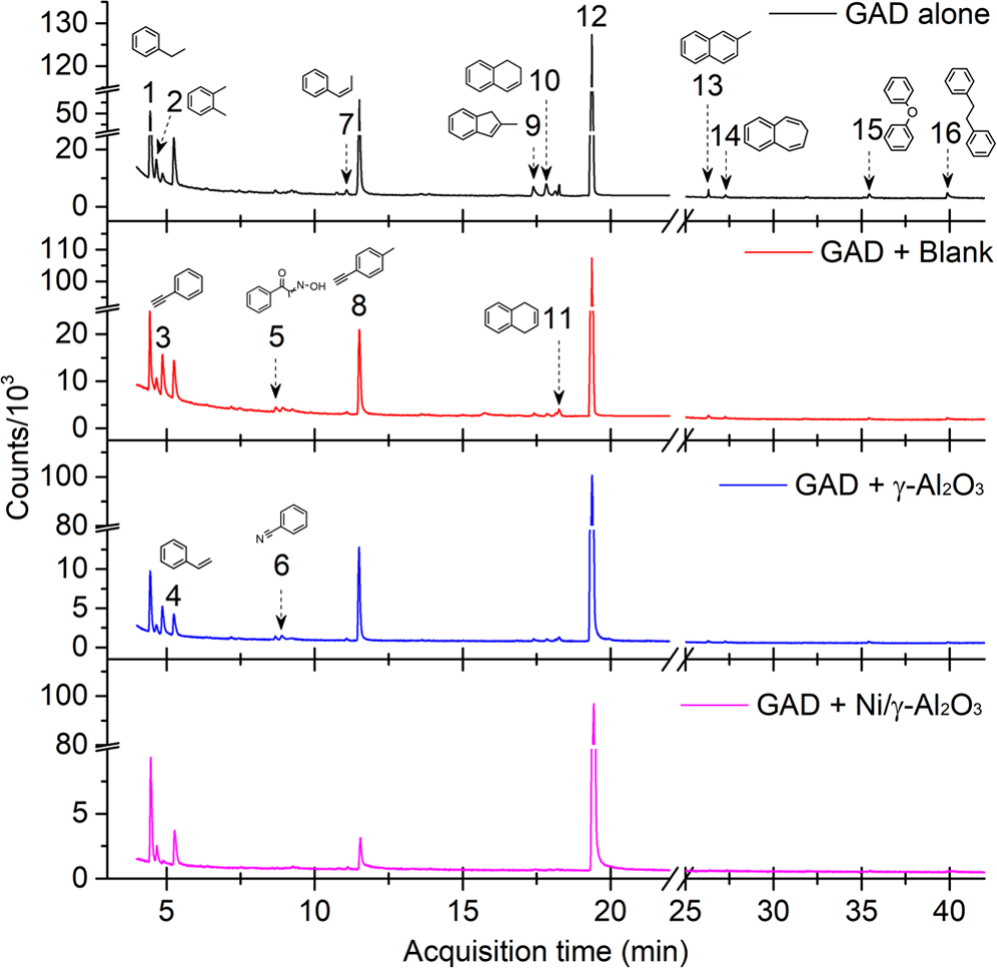
Analysis of liquid byproducts using GC-MS.

To elucidate the possible reaction pathways and underlying mechanism, liquid byproducts from different processes under the same operation condition were analyzed using GC-MS (Figure 10 and Table 1). The distribution of liquid products in the three reaction systems was quite different. Notably, the introduction of honeycomb materials into GAD narrowed the distribution of the liquid byproducts. For example, the type of liquid byproducts and their characteristic peak height were significantly decreased when using the Ni/γ-Al_2_O_3_ catalyst. These phenomena suggest that combining GAD with suitable catalysts could inhibit the accumulation of macromolecular hydrocarbons and the partial polymerization of hydrocarbon intermediates, as a variety of plasma species (e.g., electrons, OH, O, and/or N_2_*) were generated on the catalyst surface and participated in heterogeneous surface reactions to improve the degradation and oxidation of the tar compounds and their molecular fragments.^57^ Measures to further reduce the formation of byproducts should be taken from the perspectives of developing more effective and stable catalysts for biomass gasification tar reforming in plasma environments, as well as designing novel plasma-catalysis configurations to enhance the synergy between plasma discharge and catalyst.

**Table 1:** Summary of the Liquid Compounds Based on Figure 9 (Toluene is Excluded)^a^

no	chemicals	GAD alone	GAD + blank	GAD + γ-Al_2_O_3_	GAD + Ni/γ-Al_2_O_3_
1	ethylbenzene, C_8_H_10_	√√	√√	√√	√√
2	o-xylene, C_8_H_10_	√√	√	√	√
3	phenylethyne, C_8_H_6_	√	√√	√	√
4	styrene, C_8_H_8_	√√	√√	√	√
5	1-phenyl-2-nitropropene, C_9_H_9_NO_2_	√	√	√	
6	benzonitrile, C_7_H_5_N	√	√	√	
7	benzene,1-propenyl, C_9_H_10_	√	√	√	
8	benzene,1-ethynyl-4-methyl, C_9_H_8_	√√	√√	√√	√
9	1*H*-Indene,2-methyl, C_10_H_10_	√	√		
10	naphthalene,1,2-dihydro, C_10_H_10_	√√	√		
11	1,4-dihydronaphthalene, C_10_H_10_	√	√	√	
12	naphthalene, C_10_H_8_	Δ	Δ	Δ	Δ
13	naphthalene,2-methyl, C11H10	√	√		
14	benzocycloheptatriene, C_11_H_10_	√			
15	diphenyl ether, C_12_H_12_O	√			
16	bibenzyl, C_14_H_14_	√			

a √√ and Δ represent the major liquid byproducts and reactant, respectively.

**Figure 11:**
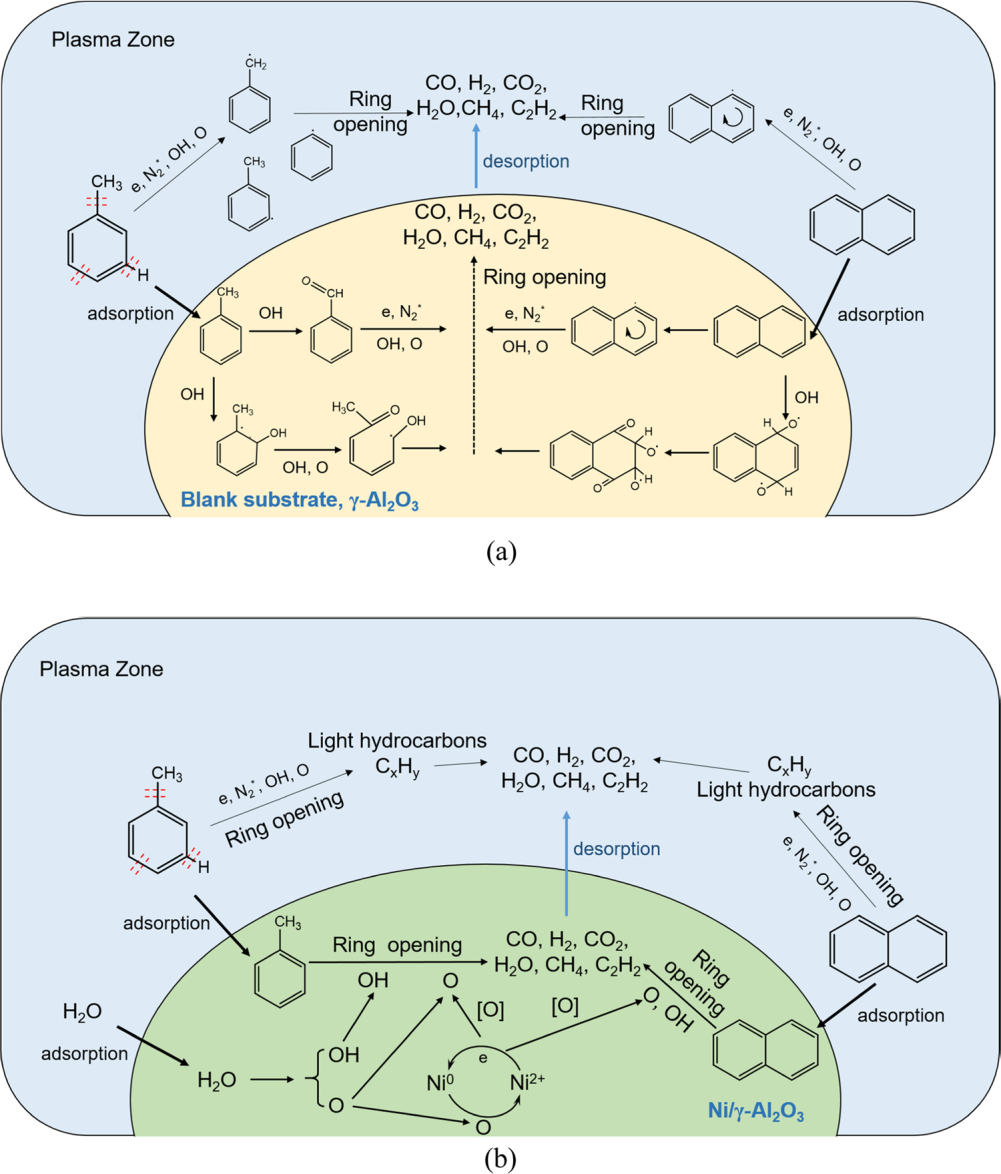
Possible reaction mechanism of tar reforming over honeycomb materials in the GAD reactor.

By reasoning and analyzing the experimental and chromatogram results as well as the catalyst characterization, the possible mechanism of tar compound conversion is proposed in Figure 11. The conversion of tar compounds in the hybrid plasma-catalytic system mainly includes three aspects: direct plasma reaction, catalytic conversion, and the synergistic effect between these two processes through the plasma-catalytic surface reaction. As discussed in the previous works, a large number of highly energetic electrons (1–10 eV) are generated in the GAD system, which could react with N_2_ and H_2_O to form the reactive species including the metastable states of N_2_*, O, and OH radicals in the gas phases (R6–R9). These reactive species then induce the degradation of toluene and naphthalene via the oxidation and ring-opening process, and form H_2_O and CO eventually.^30,32,33,38,58^
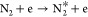
R6
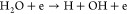
R7
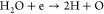
R8
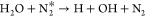
R9In the presence of the honeycomb materials (blank substrate and γ-Al_2_O_3_), the toluene and naphthalene molecules could be adsorbed on their surface to increase the probability of reacting with the plasma-generated excited species. Various aromatic hydrocarbons such as phenylethyne, benzonitrile, H-indene, 2-methyl, naphthalene, and 1,2-dihydro were then formed, and some of them experienced aromatic ring opening to generate light hydrocarbons and further converted into H_2_O, CO and CO_2_ as well as CH_4_ and C_2_H_2_, as shown in Figure 11a. When the active element Ni was loaded on the γ-Al_2_O_3_ surface, the Ni^2+^ in the metal oxide NiO was initially reduced to Ni^0^ by the energetic electrons and generated O radicals. These O radicals then react with the reactants adsorbed on the catalyst, resulting in the benzene ring opening and creating favorable conditions for the further conversion of the molecular fragments to H_2_O, CO, CO_2_, CH_4_, and C_2_H_2._^52^ These molecules finally desorbed from the catalyst surface into the gas phases (see Figure 11b). In the meantime, the H_2_O molecules could also be adsorbed onto the active sites of the catalyst and dissociated while releasing active oxygen, which oxidized the catalyst from elemental Ni^0^ to Ni^2+^. The reduction and oxidation cycle of the Ni element was continued during the plasma reforming via the facile inter-conversion between Ni^0^ and Ni^2+^ state,^27^ and maintained the stable performance of the catalyst during the steam reforming of tar using plasma catalysis.

### Performance Comparison of Different Processes for Tar Conversion

Table 2 presents the performance comparison of different processes for biomass gasification tar reforming. In thermal cracking systems, an extremely high temperature (1000 °C) was required to achieve acceptable tar conversion, while with aid of plasma discharge could significantly reduce the reaction temperature without the losses in tar conversion.^59^ Using the metal-supported catalysts lowered the temperature (600 °C–700 °C) for thermal conversion of tar and showed excellent performance.^60,61^ Plasma systems can decompose tar even at room temperature and numerous types of nonthermal plasma have shown the ability to achieve high tar conversion including DBD, GAD, and microwave plasmas.^62−65^ The hybrid plasma-catalytic systems demonstrated a higher potential to completely convert tar with high energy efficiency. Obviously, the combination of noble-metal (e.g., Rh) catalyst with plasma offered enhanced performance over Ni-based catalysts and photocatalyst (e.g., TiO_2_).^64,66^ In addition, using the honeycomb structure catalysts as in this work could decrease the overall energy consumption in the plasma process, providing a promising alternative for tar elimination. However, the tar conversion is still low and carbon deposition is detected on the used catalyst, which would negatively influence the long-term running of the plasma-catalytic system. Further investigations are still required to promote the production of syngas and the reforming efficiency while keeping a high processing capacity in the real biomass gasification conditions. The previous investigation demonstrated that the nanosecond pulsed high-voltage power source benefited the production of energetic electrons and other chemically active species for a better performance of biomass tar conversion.^66^ Using biomass char as the catalyst or support for tar conversion has received increasing interest due to its unique features of a large specific area and pore volume, high mineral content, long-term thermal stability, abundant distribution of nanoscale active clusters, and low operation cost.^67^ Therefore, approaches like developing power supply with adjustable parameters and preparing the cost-effective catalysts suitable for the hybrid plasma-catalytic systems are the directions worth working toward.

**Table 2:** Comparison of Tar Reforming Using Different Processes

process	tar surrogate	carrier gas	tar content (g/m^3^)	flow rate (m^3^/h)	conversion (%)	energy efficiency (g/kWh)	refs
thermal cracking (1000 °C)	C_8_H_10_	N_2_	1.6	0.240	100.0		(59)
plasma + thermal (800 °C)					100.0	20.5	
fixed bed + Ni/char (600 °C)	C_7_H_8_	N_2_/H_2_O	218	0.03	83.9		(60)
fixed bed + bauxite/biochar (700 °C)	C_8_H_10_	producer gas	1.6	0.023	95.0		(61)
DBD	C_7_H_8_	H_2_	33	0.0024	97.0	1.5	(62)
rotating GAD	C_7_H_8_/C_8_H_10_/C_6_H_5_OH	N_2_/H_2_O	10.0	0.360	85.7	9.5	(65)
microwave + TiO_2_	C_7_H_8_	N_2_/Ar/H_2_O	43.0	0.036	98.0	1.7	(64)
DBD + Rh/LaCoO_3_/Al_2_O_3_	C_6_H_6_/C_7_H_8_/C_10_H_8_	producer gas	10.0	0.012	100.0	25.1	(66)
DBD + Ni/γ-Al_2_O_3_	C_7_H_8_	N_2_/H_2_O	180.0	0.009	96.0	25.0	(27)
rotating GAD + Ni/γ-Al_2_O_3_	C_7_H_8_/C_8_H_10_/C_14_H_10_	N_2_/H_2_O	12.0	0.720	89.0	19.1	(63)
rotating GAD + Ni/γ-Al_2_O_3_	C_7_H_8_	N_2_/H_2_O	20.0	0.360	93.5	20.4	(39)
GAD+ Ni/γ-Al_2_O_3_ (honeycomb structure)	C_7_H_8_/C_8_H_10_	N_2_/H_2_O	16.1	0.210	85.6	50.9	this work

## Conclusions

Herein, the plasma-enhanced catalytic steam reforming of model tar compounds was performed in a GAD reactor combined with honeycomb materials. The influence of different honeycomb materials on the reaction performance was evaluated including the blank substrate as well as that coated γ-Al_2_O_3_ and Ni/γ-Al_2_O_3_. These findings indicate that introducing the honeycomb materials into the plasma environment enhanced the tar conversion and the overall energy efficiency to different extents. The best reaction performance was achieved using honeycomb material coated with Ni/γ-Al_2_O_3_, reflected by the high conversion of toluene (86.3%) and naphthalene (75.5%), the yield of H_2_ (35.0%) and CO (49.1%) and reforming efficiency (50.9 g/kWh). During the plasma-catalytic reforming, the nickel oxide species on Ni/γ-Al_2_O_3_ with a large surface area were reduced to Ni^0^ and distributed more uniformly on the support with the aid of GAD. This increased the contact and interaction between the catalyst and plasma reactive species, and generated plasma-catalysis synergy for the tar conversion with high energy efficiency and excellent catalyst stability for coke resistance. The combination of the honeycomb catalyst with GAD has shown the potential to achieve high tar conversion and acceptable energy consumption as well as attain a high yield of syngas in the gaseous products. Further investigations can focus on developing power supplies with adjustable parameters and preparing cost-effective catalysts suitable for hybrid plasma-catalytic systems.

## References

[ref1] Pawar A. U.; Kim C. W.; Nguyen-Le M.-T.; Kang Y. S. General Review on the Components and Parameters of Photoelectrochemical System for CO_2_ Reduction with in Situ Analysis. ACS Sustainable Chem. Eng. 2019, 7, 7431–7455. 10.1021/acssuschemeng.8b06303.

[ref2] Yadav K. K.; Krishnan S.; Gupta N.; Prasad S.; Amin M. A.; Cabral-Pinto M. M. S.; Sharma G. K.; Marzouki R.; Jeon B.-H.; Kumar S.; Singh N.; Kumar A.; Rezania S.; Islam S. Review on Evaluation of Renewable Bioenergy Potential for Sustainable Development: Bright Future in Energy Practice in India. ACS Sustainable Chem. Eng. 2021, 9, 16007–16030. 10.1021/acssuschemeng.1c03114.

[ref3] Zhang T. Taking on All of the Biomass for Conversion. Science 2020, 367, 1305–1306. 10.1126/science.abb1463. 32193311

[ref4] Ma W.; Chu C.; Wang P.; Guo Z.; Liu B.; Chen G. Characterization of Tar Evolution During DC Thermal Plasma Steam Gasification from Biomass and Plastic Mixtures: Parametric Optimization via Response Surface Methodology. Energy Conver. Manag. 2020, 225, 113407 10.1016/j.enconman.2020.113407.

[ref5] Anis S.; Zainal Z. A. Tar Reduction in Biomass Producer Gas Via Mechanical, Catalytic and Thermal Methods: A Review. Renewable Sustainable Energy Rev. 2011, 15, 2355–2377. 10.1016/j.rser.2011.02.018.

[ref6] Hanchate N.; Ramani S.; Mathpati C. S.; Dalvi V. H. Biomass Gasification Using Dual Fluidized Bed Gasification Systems: A Review. J. Clean. Prod. 2021, 280, 123148 10.1016/j.jclepro.2020.123148.

[ref7] Ren J.; Liu Y.-L.; Zhao X.-Y.; Cao J.-P. Biomass Thermochemical Conversion: A Review on Tar Elimination from Biomass Catalytic Gasification. J. Energy Inst. 2020, 93, 1083–1098. 10.1016/j.joei.2019.10.003.

[ref8] Kawi S.; Ashok J.; Dewangan N.; Pati S.; Chen J. Recent Advances in Catalyst Technology for Biomass Tar Model Reforming: Thermal, Plasma and Membrane Reactors. Waste Biomass Valorization 2022, 13, 1–30. 10.1007/s12649-021-01446-6.

[ref9] Ramadhani B.; Kivevele T.; Kihedu J. H.; Jande Y. A. C. Catalytic Tar Conversion and the Prospective Use of Iron-Based Catalyst in the Future Development of Biomass Gasification: A Review. Biomass Convers. Biorefin. 2022, 12, 1369–1392. 10.1007/s13399-020-00814-x.

[ref10] Huang Z.; Deng Z.; Fen Y.; Chen T.; Chen D.; Zheng A.; Wei G.; He F.; Zhao Z.; Wu J.; Li H. Exploring the Conversion Mechanisms of Toluene as a Biomass Tar Model Compound on NiFe_2_O_4_ Oxygen Carrier. ACS Sustainable Chem. Eng. 2019, 7, 16539–16548. 10.1021/acssuschemeng.9b03831.

[ref11] Galadima A.; Masudi A.; Muraza O. Catalyst Development for Tar Reduction in Biomass Gasification: Recent Progress and the Way Forward. J. Environ. Manag. 2022, 305, 114274 10.1016/j.jenvman.2021.114274. 34959056

[ref12] Du Z.-Y.; Zhang Z.-H.; Xu C.; Wang X.-B.; Li W.-Y. Low-Temperature Steam Reforming of Toluene and Biomass Tar over Biochar-Supported Ni Nanoparticles. ACS Sustainable Chem. Eng. 2019, 7, 3111–3119. 10.1021/acssuschemeng.8b04872.

[ref13] Gao N.; Salisu J.; Quan C.; Williams P. Modified Nickel-Based Catalysts for Improved Steam Reforming of Biomass Tar: A Critical Review. Renewable Sustainable Energy Rev. 2021, 145, 111023 10.1016/j.rser.2021.111023.

[ref14] Ren J.; Cao J.-P.; Yang F.-L.; Liu Y.-L.; Tang W.; Zhao X.-Y. Understandings of Catalyst Deactivation and Regeneration During Biomass Tar Reforming: A Crucial Review. ACS Sustainable Chem. Eng. 2021, 9, 17186–17206. 10.1021/acssuschemeng.1c07483.

[ref15] Wang W.; Ma Y.; Chen G.; Quan C.; Yanik J.; Gao N.; Tu X. Enhanced Hydrogen Production Using a Tandem Biomass Pyrolysis and Plasma Reforming Process. Fuel Process. Technol. 2022, 234, 107333 10.1016/j.fuproc.2022.107333.

[ref16] Gao N.; Milandile M. H.; Quan C.; Rundong L. Critical Assessment of Plasma Tar Reforming During Biomass Gasification: A Review on Advancement in Plasma Technology. J. Hazard. Mater. 2022, 421, 126764 10.1016/j.jhazmat.2021.126764. 34358972

[ref17] Chen G.; Tu X.; Homm G.; Weidenkaff A. Plasma Pyrolysis for a Sustainable Hydrogen Economy. Nat. Rev. Mater. 2022, 7, 333–334. 10.1038/s41578-022-00439-8.

[ref18] Liu L.; Zhang Z.; Das S.; Kawi S. Reforming of Tar from Biomass Gasification in a Hybrid Catalysis-Plasma System: A Review. Appl. Catal., B 2019, 250, 250–272. 10.1016/j.apcatb.2019.03.039.

[ref19] Khoja A. H.; Tahir M.; Amin N. A. S. Recent Developments in Non-Thermal Catalytic DBD Plasma Reactor for Dry Reforming of Methane. Energy Conver. Manag. 2019, 183, 529–560. 10.1016/j.enconman.2018.12.112.

[ref20] Karuppiah J.; Reddy E. L.; Reddy P. M.; Ramaraju B.; Karvembu R.; Subrahmanyam C. Abatement of Mixture of Volatile Organic Compounds (VOCs) in a Catalytic Non-Thermal Plasma Reactor. J. Hazard. Mater. 2012, 237–238, 283–289. 10.1016/j.jhazmat.2012.08.040. 22975253

[ref21] Song J.; Sima J.; Pan Y.; Lou F.; Du X.; Zhu C.; Huang Q. Dielectric Barrier Discharge Plasma Synergistic Catalytic Pyrolysis of Waste Polyethylene into Aromatics-Enriched Oil. ACS Sustainable Chem. Eng. 2021, 9, 11448–11457. 10.1021/acssuschemeng.1c03568.

[ref22] Sun Y.; Wu J.; Wang Y.; Li J.; Wang N.; Harding J.; Mo S.; Chen L.; Chen P.; Fu M.; Ye D.; Huang J.; Tu X. Plasma-Catalytic CO_2_ Hydrogenation over a Pd/ZnO Catalyst: In Situ Probing of Gas-Phase and Surface Reactions. JACS Au 2022, 10.1021/jacsau.2c00028. PMC9400056 36032530

[ref23] Mei D.; Zhu X.; Wu C.; Ashford B.; Williams P. T.; Tu X. Plasma-Photocatalytic Conversion of CO_2_ at Low Temperatures: Understanding the Synergistic Effect of Plasma-Catalysis. Appl. Catal., B 2016, 182, 525–532. 10.1016/j.apcatb.2015.09.052.

[ref24] Zhu X.; Gao X.; Qin R.; Zeng Y.; Qu R.; Zheng C.; Tu X. Plasma-Catalytic Removal of Formaldehyde over Cu–Ce Catalysts in a Dielectric Barrier Discharge Reactor. Appl. Catal., B 2015, 170–171, 293–300. 10.1016/j.apcatb.2015.01.032.

[ref25] Wu Z.; Zhu Z.; Hao X.; Zhou W.; Han J.; Tang X.; Yao S.; Zhang X. Enhanced Oxidation of Naphthalene Using Plasma Activation of TiO_2_/Diatomite Catalyst. J. Hazard. Mater. 2018, 347, 48–57. 10.1016/j.jhazmat.2017.12.052. 29289765

[ref26] Ronda-Lloret M.; Wang Y.; Oulego P.; Rothenberg G.; Tu X.; Shiju N. R. CO_2_ Hydrogenation at Atmospheric Pressure and Low Temperature Using Plasma-Enhanced Catalysis over Supported Cobalt Oxide Catalysts. ACS Sustainable Chem. Eng. 2020, 8, 17397–17407. 10.1021/acssuschemeng.0c05565. 33282570 PMC7709469

[ref27] Liu L.; Wang Q.; Ahmad S.; Yang X.; Ji M.; Sun Y. Steam Reforming of Toluene as Model Biomass Tar to H_2_-Rich Syngas in a DBD Plasma-Catalytic System. J. Energy Inst. 2018, 91, 927–939. 10.1016/j.joei.2017.09.003.

[ref28] Liu S. Y.; Mei D. H.; Nahil M. A.; Gadkari S.; Gu S.; Williams P. T.; Tu X. Hybrid Plasma-Catalytic Steam Reforming of Toluene as a Biomass Tar Model Compound over Ni/Al_2_O_3_ Catalysts. Fuel Process. Technol. 2017, 166, 269–275. 10.1016/j.fuproc.2017.06.001.

[ref29] Xu B.; Wang N.; Xie J.; Song Y.; Huang Y.; Yang W.; Yin X.; Wu C. Removal of Toluene as a Biomass Tar Surrogate by Combining Catalysis with Nonthermal Plasma: Understanding the Processing Stability of Plasma Catalysis. Catal. Sci. Technol. 2020, 10, 6953–6969. 10.1039/d0cy01211d.

[ref30] Mei D.; Wang Y.; Liu S.; Alliati M.; Yang H.; Tu X. Plasma Reforming of Biomass Gasification Tars Using Mixed Naphthalene and Toluene as Model Compounds. Energy Conver. Manag. 2019, 195, 409–419. 10.1016/j.enconman.2019.05.002.

[ref31] Wang Y.; Liao Z.; Mathieu S.; Bin F.; Tu X. Prediction and Evaluation of Plasma Arc Reforming of Naphthalene Using a Hybrid Machine Learning Model. J. Hazard. Mater. 2021, 404, 123965 10.1016/j.jhazmat.2020.123965. 33017710

[ref32] Liu S.; Mei D.; Wang L.; Tu X. Steam Reforming of Toluene as Biomass Tar Model Compound in a Gliding Arc Discharge Reactor. Chem. Eng. J. 2017, 307, 793–802. 10.1016/j.cej.2016.08.005.

[ref33] Zhang H.; Zhu F.; Li X.; Xu R.; Li L.; Yan J.; Tu X. Steam Reforming of Toluene and Naphthalene as Tar Surrogate in a Gliding Arc Discharge Reactor. J. Hazard. Mater. 2019, 369, 244–253. 10.1016/j.jhazmat.2019.01.085. 30780020

[ref34] Mei D.; Zhang P.; Liu S.; Ding L.; Ma Y.; Zhou R.; Gu H.; Fang Z.; Cullen P. J.; Tu X. Highly Efficient Reforming of Toluene to Syngas in a Gliding Arc Plasma Reactor. J. Energy Inst. 2021, 98, 131–143. 10.1016/j.joei.2021.06.005.

[ref35] Zhang H.; Li L.; Xu R.; Huang J.; Wang N.; Li X.; Tu X. Plasma-Enhanced Catalytic Activation of CO_2_ in a Modified Gliding Arc Reactor. Waste Dispos. Sustainable Energy 2020, 2, 139–150. 10.1007/s42768-020-00034-z.

[ref36] Zhu F.; Zhang H.; Yan X.; Yan J.; Ni M.; Li X.; Tu X. Plasma-Catalytic Reforming of CO_2_-Rich Biogas over Ni/γ-Al_2_O_3_ Catalysts in a Rotating Gliding Arc Reactor. Fuel 2017, 199, 430–437. 10.1016/j.fuel.2017.02.082.

[ref37] Zhang H.; Tan Q.; Huang Q.; Wang K.; Tu X.; Zhao X.; Wu C.; Yan J.; Li X. Boosting the Conversion of CO_2_ with Biochar to Clean CO in an Atmospheric Plasmatron: A Synergy of Plasma Chemistry and Thermochemistry. ACS Sustainable Chem. Eng. 2022, 10, 7712–7725. 10.1021/acssuschemeng.2c01778.

[ref38] Mei D.; Liu S.; Wang Y.; Yang H.; Bo Z.; Tu X. Enhanced Reforming of Mixed Biomass Tar Model Compounds Using a Hybrid Gliding Arc Plasma Catalytic Process. Catal. Today 2019, 337, 225–233. 10.1016/j.cattod.2019.05.046.

[ref39] Xu R.; Kong X.; Zhang H.; Ruya P. M.; Li X. Destruction of Gasification Tar over Ni Catalysts in a Modified Rotating Gliding Arc Plasma Reactor: Effect of Catalyst Position and Nickel Loading. Fuel 2021, 289, 119742 10.1016/j.fuel.2020.119742.

[ref40] Bogaerts A.; Tu X.; Whitehead J. C.; Centi G.; Lefferts L.; Guaitella O.; Azzolina-Jury F.; Kim H.-H.; Murphy A. B.; Schneider W. F.; Nozaki T.; Hicks J. C.; Rousseau A.; Thevenet F.; Khacef A.; Carreon M. The 2020 Plasma Catalysis Roadmap. J. Phys. D: Appl. Phys. 2020, 53, 443001 10.1088/1361-6463/ab9048.

[ref41] Hossain M. M.; Mok Y. S.; Nguyen D. B.; Kim S. J.; Kim Y. J.; Lee J. H.; Heo I. Nonthermal Plasma in Practical-Scale Honeycomb Catalysts for the Removal of Toluene. J. Hazard. Mater. 2021, 404, 123958 10.1016/j.jhazmat.2020.123958. 33068994

[ref42] Ashford B.; Wang Y.; Poh C. K.; Chen L.; Tu X. Plasma-Catalytic Conversion of CO_2_ to CO over Binary Metal Oxide Catalysts at Low Temperatures. Appl. Catal., B 2020, 276, 119110 10.1016/j.apcatb.2020.119110.

[ref43] Hemra K.; Aungkavattana P. Effect of Cordierite Addition on Compressive Strength and Thermal Stability of Metakaolin Based Geopolymer. Adv. Powder Technol. 2016, 27, 1021–1026. 10.1016/j.apt.2016.04.019.

[ref44] Han F.; Liu H.; Cheng W.; Xu Q. Highly Selective Conversion of CO_2_ to Methanol on the Cuzno-ZrO_2_ Solid Solution with the Assistance of Plasma. RSC Adv. 2020, 10, 33620–33627. 10.1039/d0ra00961j. 35519065 PMC9056770

[ref45] Sun S.; Li H.; Xu Z. J. Impact of Surface Area in Evaluation of Catalyst Activity. Joule 2018, 2, 1024–1027. 10.1016/j.joule.2018.05.003.

[ref46] Wu Y.-W.; Chung W.-C.; Chang M.-B. Modification of Niγ-Al_2_O_3_ Catalyst with Plasma for Steam Reforming of Ethanol to Generate Hydrogen. Int. J. Hydrogen Energy 2015, 40, 8071–8080. 10.1016/j.ijhydene.2015.04.053.

[ref47] Nguyen V. T.; Nguyen D. B.; Mok Y. S.; Hossain M. M.; Saud S.; Yoon K. H.; Dinh D. K.; Ryu S.; Jeon H.; Kim S. B. Removal of Ethyl Acetate in Air by Using Different Types of Corona Discharges Generated in a Honeycomb Monolith Structure Coated with Pd/Gamma-Alumina. J. Hazard. Mater. 2021, 416, 126162 10.1016/j.jhazmat.2021.126162. 34492940

[ref48] Xiao K.; Li X.; Santoso J.; Wang H.; Zhang K.; Wu J.; Zhang D. Synergistic Effect of Dielectric Barrier Discharge Plasma and Mn Catalyst on CO_2_ Reforming of Toluene. Fuel 2021, 285, 119057 10.1016/j.fuel.2020.119057.

[ref49] Azalim S.; Brahmi R.; Agunaou M.; Beaurain A.; Giraudon J. M.; Lamonier J. F. Washcoating of Cordierite Honeycomb with Ce–Zr–Mn Mixed Oxides for VOC Catalytic Oxidation. Chem. Eng. J. 2013, 223, 536–546. 10.1016/j.cej.2013.03.017.

[ref50] Belbessai S.; Achouri I. E.; Benyoussef E. H.; Gitzhofer F.; Abatzoglou N. Toluene Steam Reforming Using Nickel Based Catalysts Made from Mining Residues. Catal. Today 2021, 365, 111–121. 10.1016/j.cattod.2020.07.087.

[ref51] Zhu D.; Chen Z.; Li J.; Wu Z.; Gao E.; Wang W.; Yao S. Evaluation of Au/Gamma-Al_2_O_3_ Nanocatalyst for Plasma-Catalytic Decomposition of Toluene. Chemosphere 2021, 285, 131474 10.1016/j.chemosphere.2021.131474. 34329130

[ref52] Lu M.; Yang W.; Yu C.; Liu Q.; Ye D. Plasma-Catalytic Oxidation of Toluene on Ag Modified FeO_x_/SBA-15. Aerosol Air Qual. Res. 2020, 20, 193–202. 10.4209/aaqr.2019.09.0467.

[ref53] Mlotek M.; Ulejczyk B.; Woroszyl J.; Krawczyk K. Decomposition of Toluene in Coupled Plasma-Catalytc System. Ind. Eng. Chem. Res. 2020, 59, 4239–4244. 10.1021/acs.jecr.9b04330.

[ref54] Cimerman R.; Račková D.; Hensel K. Tars Removal by Non-Thermal Plasma and Plasma Catalysis. J. Phys. D: Appl. Phys. 2018, 51, 274003 10.1088/1361-6463/aac762.

[ref55] Lee H.; Sekiguchi H. Plasma-Catalytic Hybrid System Using Spouted Bed with a Gliding Arc Discharge: CH_4_ reforming as a Model Reaction. J. Phys. D: Appl. Phys. 2011, 44, 274008 10.1088/0022-3727/44/27/274008.

[ref56] Al-Fatesh A. S.; Arafat Y.; Atia H.; Ibrahim A. A.; Ha Q. L. M.; Schneider M.; M-Pohl M.; Fakeeha A. H. CO_2_-Reforming of Methane to Produce Syngas over Co-Ni/SBA-15 Catalyst: Effect of Support Modifiers (Mg, La and Sc) on Catalytic Stability. J. CO2 Util. 2017, 21, 395–404. 10.1016/j.jcou.2017.08.001.

[ref57] Wu K.; Sun Y.; Liu J.; Xiong J.; Wu J.; Zhang J.; Fu M.; Chen L.; Huang H.; Ye D. Nonthermal Plasma Catalysis for Toluene Decomposition over BaTiO_3_-Based Catalysts by Ce Doping at A-Sites: The Role of Surface-Reactive Oxygen Species. J. Hazard. Mater. 2021, 405, 124156 10.1016/j.jhazmat.2020.124156. 33246817

[ref58] Wang Y.; Yang H.; Tu X. Plasma Reforming of Naphthalene as a Tar Model Compound of Biomass Gasification. Energy Conver. Manag. 2019, 187, 593–604. 10.1016/j.enconman.2019.02.075.

[ref59] Gomez-Rueda Y.; Zaini I. N.; Yang W.; Helsen L. Thermal Tar Cracking Enhanced by Cold Plasma - a Study of Naphthalene as Tar Surrogate. Energy Conver. Manag. 2020, 208, 112540 10.1016/j.enconman.2020.112540.

[ref60] Ren L.; Yan L.-J.; Bai Y.-H.; Liu Y.; Lv P.; Wang Y.-X.; Li F. Effects of Loading Methods and Oxidation Degree of Support on the Tar Reforming Activity of Char-Supported Ni Catalyst Using Toluene as a Model Compound. Fuel Process. Technol. 2020, 201, 106347 10.1016/j.fuproc.2020.106347.

[ref61] Cheng L.; Wu Z.; Zhang Z.; Guo C.; Ellis N.; Bi X.; Paul Watkinson A.; Grace J. R. Tar Elimination from Biomass Gasification Syngas with Bauxite Residue Derived Catalysts and Gasification Char. Appl. Energy 2020, 258, 114088 10.1016/j.apenergy.2019.114088.

[ref62] Saleem F.; Harvey A.; Zhang K. Low Temperature Conversion of Toluene to Methane Using Dielectric Barrier Discharge Reactor. Fuel 2019, 248, 258–261. 10.1016/j.fuel.2019.02.137.

[ref63] Kong X.; Zhang H.; Li X.; Xu R.; Mubeen I.; Li L.; Yan J. Destruction of Toluene, Naphthalene and Phenanthrene as Model Tar Compounds in a Modified Rotating Gliding Arc Discharge Reactor. Catalysts 2019, 9, 19 10.3390/catal9010019.

[ref64] Sun J.; Wang Q.; Wang W.; Wang K. Study on the Synergism of Steam Reforming and Photocatalysis for the Degradation of Toluene as a Tar Model Compound under Microwave-Metal Discharges. Energy 2018, 155, 815–823. 10.1016/j.energy.2018.05.045.

[ref65] Xu R.; Zhu F.; Zhang H.; Ruya P. M.; Kong X.; Li L.; Li X. Simultaneous Removal of Toluene, Naphthalene, and Phenol as Tar Surrogates in a Rotating Gliding Arc Discharge Reactor. Energy Fuels 2020, 34, 2045–2054. 10.1021/acs.energyfuels.9b03529.

[ref66] Dors M.; Kurzyńska D. Tar Removal by Nanosecond Pulsed Dielectric Barrier Discharge. Appl. Sci. 2020, 10, 991 10.3390/app10030991.

[ref67] Chen X.; Ma X.; Peng X.; Chen L.; Lu X.; Tian Y. Effect of Synthesis Temperature on Catalytic Activity and Coke Resistance of Ni/Bio-Char During CO_2_ Reforming of Tar. Int. J. Hydrog. Energy 2021, 46, 27543–27554. 10.1016/j.ijhydene.2021.06.011.

